# Phytoestrogen Concentrations in Human Urine as Biomarkers for Dietary Phytoestrogen Intake in Mexican Women

**DOI:** 10.3390/nu9101078

**Published:** 2017-09-29

**Authors:** Karina M. Chávez-Suárez, María I. Ortega-Vélez, Ana I. Valenzuela-Quintanar, Marcia Galván-Portillo, Lizbeth López-Carrillo, Julián Esparza-Romero, María S. Saucedo-Tamayo, María R. Robles-Burgueño, Susana A. Palma-Durán, María L. Gutiérrez-Coronado, Melissa M. Campa-Siqueiros, Patricia Grajeda-Cota, Graciela Caire-Juvera

**Affiliations:** 1 Department of Nutrition, Section of Public Nutrition and Health, Centro de Investigación en Alimentación y Desarrollo, A.C. (CIAD), Carretera a La Victoria km 0.6, 83304 Hermosillo, Sonora, Mexico; kamachasu@gmail.com (K.M.C.-S.); iortega@ciad.mx (M.I.O.-V.); julian@ciad.mx (J.E.-R.); coco@ciad.mx (M.S.S.-T.); melita379@hotmail.com (M.M.C.-S.); 2 Department of Food Sciences, Centro de Investigación en Alimentación y Desarrollo, A.C. (CIAD), Carretera a La Victoria km 0.6, 83304 Hermosillo, Sonora, Mexico; aquintanar@ciad.mx (A.I.V.-Q.); cuquis@ciad.mx (M.R.R.-B.); sussypalmaa@gmail.com (S.A.P.-D.); lulu@ciad.mx (M.L.G.-C.); grajeda@ciad.mx (P.G.-C.); 3 Center for Population Health Research, Instituto Nacional de Salud Pública, Universidad No. 655, Colonia Santa María Ahuacatitlán, Cerrada Los Pinos y Caminera, 62100 Cuernavaca, Morelos, Mexico; mgalvan@insp.mx (M.G.-P.); lizbeth@insp.mx (L.L.-C.)

**Keywords:** phytoestrogen intake, urinary excretion, biomarker, Mexican women

## Abstract

There has been substantial interest in phytoestrogens, because of their potential effect in reducing cancer and heart disease risk. Measuring concentrations of phytoestrogens in urine is an alternative method for conducting epidemiological studies. Our objective was to evaluate the urinary excretion of phytoestrogens as biomarkers for dietary phytoestrogen intake in Mexican women. Participants were 100 healthy women from 25 to 80 years of age. A food frequency questionnaire (FFQ) and a 24 h recall were used to estimate habitual and recent intakes of isoflavones, lignans, flavonols, coumestrol, resveratrol, naringenin, and luteolin. Urinary concentrations were measured by liquid chromatography (HPLC) coupled to mass spectrometry (MS) using the electrospray ionization interface (ESI) and diode array detector (DAD) (HPLC-DAD-ESI-MS). Spearman correlation coefficients were used to evaluate associations between dietary intake and urine concentrations. The habitual consumption (FFQ) of total phytoestrogens was 37.56 mg/day. In urine, the higher compounds were naringenin (60.1 µg/L) and enterolactone (41.7 µg/L). Recent intakes (24 h recall) of isoflavones (*r* = 0.460, *p* < 0.001), lignans (*r* = 0.550, *p* < 0.0001), flavonoids (*r* = 0.240, *p* < 0.05), and total phytoestrogens (*r* = 0.410, *p* < 0.001) were correlated to their urinary levels. Total phytoestrogen intakes estimated by the FFQ showed higher correlations to urinary levels (*r* = 0.730, *p* < 0.0001). Urinary phytoestrogens may be useful as biomarkers of phytoestrogen intake, and as a tool for evaluating the relationship of intake and disease risk in Mexican women.

## 1. Introduction

Experimental evidence suggests that phytoestrogen intake may modulate the risk of cancer and cardiovascular disease [1,2,3,4]. Phytoestrogens are plant-derived, naturally occurring non-steroidal polycyclic phenols that may have weak estrogenic effects when they are ingested and metabolized [5]. Major classes of phytoestrogens are isoflavones, lignans, and coumestans. Diet is the main source of phytoestrogens in humans. Isoflavones are present in berries, soybeans, and other legumes [6]. Lignans, primarily matairesinol and secoisolariciresinol, are found widely in many fiber rich foods such as fruits, vegetables, cereals, and flaxseeds [7]. The main dietary sources of coumestans are alfalfa sprouts, followed by pinto and pea beans [8]. Resveratrol, from the stilbene group, is found in wine, grape skins, and peanuts [9]. 

The estimation of dietary consumption of phytoestrogens is limited by the scarcity of data on the content of food; therefore, food composition databases are complex to establish. Phytoestrogen analysis in food is complicated due to the variety of matrices and the different concentrations in foods. Thus, there is a need for a validated biomarker of phytoestrogen intake for epidemiological studies [10]. An alternative approach for determining the intake of phytoestrogens, is measuring them in biological samples, such as urine. 

Phytoestrogens in food are mainly in the form of several types of conjugates (β-glycosidic) and in smaller amounts as aglycones. Conjugate forms (inactive) are ingested and hydrolyzed to their aglycone forms (bioactive) by bacterial β-glucosidases in the intestine wall. Then, only the bioactive forms are absorbed by the intestinal tract, and can be further glucuronidated in the intestinal wall and liver [11]. Overall, the main circulating and excreting forms of phytoestrogens are the glucuronidated metabolites. Urinary excretion of phytoestrogens varies with the type of diet, which may be related to differences in both human pharmacokinetics and metabolism by the intestinal bacteria [12]. 

At population level, little information is available on the excretion of urinary phytoestrogens and their associations with dietary intakes in Western countries. Dietary intake of phytoestrogens has been associated with urinary excretion of related metabolites in observational studies [1,13,14,15] and controlled trials [16,17]. In Mexico, a study evaluated the phytoestrogens consumption in typical diets [18]; however, a main limitation was the scarcity of foods with phytoestrogen concentrations in food composition databases. We have previously evaluated phytoestrogens in serum as biomarkers of intake in Mexican women, although limited correlations for individual compounds were found [19]. Furthermore, there are no studies on the concentration of urinary phytoestrogens of the general population. Therefore, the ability to monitor objectively exposure to these compounds is important in order to understand the health impact of dietary intake of phytoestrogens in Mexican population. A better understanding of the relationship of phytoestrogen consumption and urine concentration in the population of Mexico is essential for carrying out epidemiological research regarding the association between phytoestrogens and health. 

Therefore, the aim of this study was to determine phytoestrogens in urine as biomarkers of phytoestrogen intake, by measuring the association of urinary excretion of these compounds with recent and habitual dietary intakes in Mexican women. Estimates of dietary intakes of isoflavones (genistein, daidzein, equol, glycitein, formononetin, biochanin A), lignans (enterodiol, enterolactone, secoisolariciresinol, matairesinol), flavonols (quercetin, kaempferol), flavanones (naringenin), flavones (luteolin), stilbenes (resveratrol), and coumestans (coumestrol) were compared with their urinary compounds. 

## 2. Materials and Methods

### 2.1. Study Subjects

Subjects in this cross-sectional study were 100 apparently healthy women, aged 25 to 80 years and with at least 5 years of residence in Northwest México. Women were excluded from the study if they were pregnant or breastfeeding, or have had prior diagnosis of specific chronic illnesses such as diabetes, cancer, or heart disease. Participants were selected randomly from 20 blocks in the city of Hermosillo, Sonora, Mexico, through the Master Sample Frame, which is the basis sample of houses for the surveys that make up the System of National Health Surveys in México.

The protocol for this research was reviewed and approved by the Ethical Committee from Centro de Investigación en Alimentación y Desarrollo (Center for Research on Food and Development). The investigation was carried out following the Declaration of Helsinki; all women provided written informed consent prior to participation. Socioeconomic and demographic indicators were collected at first interview in women’s households. Weight and height were measured using standardized procedures to characterize the study population. Weight was measured by a portable electronic scale (and FG-150KBM (up to 150 kg weighing capacity) with 0.05 kg of accuracy). Height was measured using a portable stadiometer (SECA). Body mass index was calculated from weight and height.

### 2.2. Dietary Assessment

Habitual intake of phytoestrogens was assessed using a food frequency questionnaire (FFQ) validated for women in the region [20] and modified for this study. The questionnaire was applied by trained interviewers, and included 162 food items from 11 categories. Frequency of consumption of each food item was evaluated on a daily, weekly, or monthly basis, and portion sizes (small, medium, large) were specified. 

Recent intake of phytoestrogens was assessed using a 24 h dietary recall on the same day that the urine sample was taken. Printed food models, plates, glasses and spoons were used as visual aids to improve recall. Additionally, a second 24 h dietary recall was applied to a subsample of 50 women, with a minimum of three weeks between interviews, to calculate the coefficient of intraindividual variation of daily intake.

Quantification of daily intakes of 16 phytoestrogens considered in this study was performed using a food dictionary that included data from the USDA [6], the National Institute of Nutrition in Mexico, and the food composition database from our Institute. In addition, tables and databases from different studies [7,8,9,21,22,23,24] were used to identify foods that are a source of phytoestrogens. Total phytoestrogen intake was calculated as the sum of lignans, isoflavones, flavonoids, coumestrol and resveratrol intakes.

### 2.3. Urine Collection 

Subjects were provided with supplies and instructed in the total collection of 12 h urine on any day but those of the menstrual period. Participants should not have consumed any antibiotics or other kind of drugs within 7 days prior to urine collection. Total 12 h urine was collected in 0.5 L portable containers with 0.5 g of *L*-ascorbic acid (A4544-100G, Sigma-Aldrich, St. Louis, MO, USA), to prevent microbial contamination and oxidative degradation [25]. Subjects were asked to use a separate container for before-dinner urine, overnight urine, and the first urine excreted in the morning of the following day, and to store containers in their refrigerators at 4–6 °C or cooler until the next morning. Total 12 h urine samples were picked up and transported in a cooler to the laboratory. The total urine volume of each subject was measured, and a sample was filtered using Whatman grade 41 filters. The filtered samples were dispensed into 5 mL aliquots and stored in polypropylene cryogenic vials at −70 °C until analysis of phytoestrogens. One aliquot was sent to a biomedical laboratory for analysis of urinary creatinine.

### 2.4. Urine Analysis

Urine samples (*n* = 100) were analyzed for phytoestrogens using liquid–liquid extraction (LLE) based on a protocol published by Bolca et al. [26], followed by a high-resolution chromatography-mass spectrometry (HPLC-MS) analysis using the method described by Wyns et al. [27], previously modified for the analysis of phytoestrogens using HPLC-DAD-ESI-mass spectrometry. Urine samples were analyzed for total concentration of isoflavones (genistein, daidzein, equol, glicitein, formononetin, biochanine A), lignans (enterodiol, enterolactone, secoisolariciresinol, matairesinol), coumestans (coumestrol), stilbenes (resveratrol), flavonols (quercetin, kaempferol), flavanones (naringenin), and flavones (luteolin). Quercetin, luteolin, and naringenin were obtained from Indofine Chemical (Hillsborough, NJ, USA). The remaining phytoestrogens, the internal standard (4-hydroxybenzophenone), deconjugation internal standards (β-phenolphthalein glucuronide and 4-methylumbelliferone sulfate), and *Helix pomatia*, H1, were obtained from Sigma-Aldrich. 

Prior to extraction, urine samples were centrifuged at 3000 rpm for 10 min to remove solids and 2.0 mL of supernatant were diluted with 2.0 mL of sodium acetate buffer (pH 5.0; 0.1 M), spiked with 100 µL of internal standard 4-hydroxybenzophenone (4 µg/mL) and 10 µL of deconjugation internal standards (20 µg/mL). Conjugated analytes were hydrolyzed by the addition of 30 µL β-glucuronidase/sulfatase (*Helix pomatia*, H1, containing 5.14 mg of enzyme in 1 mL of sodium acetate buffer, pH 5.0, 0.1 M) and incubated 4 h at 37 °C. Urine-deconjugated samples were extracted (twice) with 5 mL of diethyl ether and subsequent vortex mixing (30 s). After samples stand for 5 min, the organic phase, containing phytoestrogens, was transferred into a new 15 mL conical tube. Eluates collected were dried under a gentle nitrogen stream, dissolved in a 200 µL injection solvent (40/60, *v*/*v*) (initial mobile phase (65A:35B) and methanol) with a gentle vortexing for 1 min and transferred into an amber vial for HPLC-DAD-ESI analysis. The phytoestrogens in the extracted samples were separated by injecting 50 µL of filtered extract into the Agilent 1100 series HPLC system (Agilent Technologies Inc., Palo Alto, CA, USA). 

HPLC analyses were carried out using a Waters XBridge C18 reversed phase (3.5 µm) column (3.0 mm internal diameter × 150 mm) connected to a C18 guard column (3.0 mm internal diameter × 20 mm) and maintained at a temperature of 52 °C. The mobile phase was milliQ H_2_O (Solvent A) and MeOH/CH_3_CN (80/20, *w*/*w*) (Solvent B), both acidified with 0.025% (*v*/*v*) formic acid. The gradient and flow rate were programmed as follows: 0–5.0 min: 35–40% B (flow: 0.6 mL/min), 5.0–16.0 min: 40–100% B (flow: 0.6 mL/min), 16.0–19.0 min: isocratic 100% B (flow. 0.8 mL/min), 19.0–19.1 min: 100–35% B (flow: 0.6 mL/min) and 2.9 min for re-equilibration of the column before subsequent injection. Analyses were monitored simultaneously by diode array detection at 260, 280, 290, 310, and 360 nm during the entire HPLC run. All sample compounds were ionized by electrospray (ESI) in the negative ion mode and analyzed by single quadrupole mass spectrometry for confirmation of phytoestrogens. The drying temperature was 350 °C. The nebulizer pressure was 60 psi, the capillary voltage 3.5 kV, and the drying gas flow 11 L/min. Quality control samples (spiked urine) were analyzed along with unknown samples. 

Linearity of the method (*r*^2^ = 0.995–0.999) was established using eight different concentrations covering from 0.16 to 2400 ng/mL. The limit of detection was from 0.001 to 0.075 ng/mL. In addition, different blanks were run to exclude the presence of interferences and ensure the results. The recovery of phytoestrogens was from 80 to 120%, with the exception of resveratrol (50%) and secoisolarisiresinol (60%). 

### 2.5. Statistical Analysis

Urinary concentrations of phytoestrogens are expressed as micrograms of phytoestrogens per gram of urinary creatinine (µg/g) by adjusting concentrations for urinary creatinine levels [26]. The mean dietary nutrient intake was adjusted for total energy intake using the residual method [28]. Spearman rank correlations were used to assess the relationship of habitual and recent intake of total phytoestrogens and subgroups with urinary phytoestrogen excretion. The raw correlation was first evaluated and then attenuated considering intraindividual variation in daily consumption of food. Statistical analyses were performed using the STATA statistical software package, version 8.0 (StataCorp LP, College Station, TX, USA). Statistical significance was assumed at the level of 0.05.

## 3. Results

### 3.1. General Characteristics of Participants

Mean age of the women was 44.7 ± 12.7 years. The majority of women were premenopausal (57%), married (74%), and of low socioeconomic status (62%). Overall, the prevalence of overweight and obesity according to body mass index was 79%. When nutrient intakes were estimated by the 24 h recall, mean energy intake was 1473 kcal/day, and, by the FFQ, energy intake was 2563 kcal/day.

### 3.2. Intake of Phytoestrogens

There was a wide variation in intake among phytoestrogen groups, values were right-skewed. The mean and geometric mean of phytoestrogens intake estimated by the FFQ are shown in Table 1 and Table 2. Intakes of naringenin, quercetin, and coumestrol were the highest. The more consumed isoflavone was genistein, followed by daidzein. On the other hand, estimation of phytoestrogen intakes using the 24 h recall (Table 3 and Table 4) showed that naringenin, quercetin, and genistein were the most consumed phytoestrogens. Recent intake of coumestrol (403 µg/day) was lower than its estimated habitual intake (1850 µg/day). In general, estimated recent intakes of phytoestrogens were lower than habitual intakes.

### 3.3. Phytoestrogens in Urine

Urine volumes ranged from 0.22 to 1.7 L, and the mean concentration of creatinine was 0.63 g/L. The majority of phytoestrogens (75%) were detected in 77% of the urine samples analyzed. Table 5 shows that, from the group of isoflavones, biochanin A and daidzein had respectively the lowest and highest concentrations in urine. From the group of lignans, enterolactone was the more detected phytoestrogen in urine, and the lower concentration was found for matairesinol, which is metabolized to enterolactone. In the flavonoid group, naringenin in urine was 40 times higher than quercetin.

### 3.4. Correlations of Dietary and Urinary Phytoestrogens

The corrected correlation coefficients between phytoestrogen intakes estimated by the FFQ and by the 24 h recall and their concentrations in urine are shown in Table 6. Using the FFQ, only dietary and urinary resveratrol were correlated (*r* = 0.337, *p* < 0.01). Although at the individual level correlations were not found between habitual dietary intakes (FFQ) and urine concentrations, total phytoestrogens showed a high correlation (*r* = 0.730, *p* < 0.001) between FFQ and urine excretion. On the other hand, recent intakes (24 h recall) of genistein (*r* = 0.374, *p* < 0.01), and naringenin (*r* = 0.620, *p* < 0.0001) were correlated with their respective urinary levels. As a group, recent consumptions (24 h recall) of isoflavones (*r* = 0.460, *p* < 0.001), lignans (*r* = 0.550, *p* < 0.0001), flavonoids (*r* = 0.240, *p* < 0.05), and total phytoestrogens (*r* = 0.410, *p* < 0.001) correlated with their urinary excretion.

Enterodiol and matairesinol were detected in only 15% and 26% of the urine samples. Spearman’s correlation coefficient between urinary daidzein concentration and its metabolite equol was 0.30 (*p* = 0.002). Additionally, urinary secoisolariciresinol concentration was correlated with its metabolite enterodiol (*r* = 0.23; *p* = 0.019). 

## 4. Discussion

In this study, we estimated habitual and recent dietary intakes of phytoestrogens, and measured the urinary excretion of these compounds and related metabolites in a group of Mexican adult women. We also evaluated the relationship between dietary and urinary phytoestrogens and observed that total dietary phytoestrogens, estimated by the FFQ, correlated to their urinary excretion. When we estimated dietary intake of phytoestrogens using the 24 h dietary recall, more correlations between dietary and urinary phytoestrogens were found, including genistein, naringenin, isoflavones, lignans, flavonoids, and total phytoestrogens.

Total phytoestrogens in our study included 16 individual compounds. No observational studies have examined simultaneously a wide range of phytoestrogens. The degree of correlation observed between total urinary phytoestrogens and our estimates of dietary intake were in the magnitude of 0.73 (corrected) for the FFQ, and 0.41 (corrected) for the 24 h recall. Correlations for the FFQ are higher than those obtained in previous study where the correlation between total dietary intake and urinary phytoestrogens was 0.54 [29]. As observed, we obtained a higher correlation of total phytoestrogens using the FFQ, compared to the 24 h recall. This may be because the composition of phytoestrogens in certain food items that were consumed in the previous 24 h were not included in the food database; some phytoestrogens have not been analyzed for particular food components. Since total phytoestrogens consist of the sum of all the individual compounds, the lack of one or more of them contributes to a lower concentration of total phytoestrogens. On the other hand, the FFQ contains a larger list of foods, and many of them may have a complete composition of phytoestrogens in the database.

Some authors attribute the weak or null association of individual compounds to the extensive response categories used in the FFQ, and imprecise interpretation of the interviewer to describe “a few times a week” or “daily or almost daily” [30]. Additionally, composition tables used are not representative of the foods that were consumed. Thus, dietary intake data in our study were based on estimated values and were therefore less accurate than intakes measured by urinary concentrations.

The information available on the dietary intake of phytoestrogens in the general population in Western countries is limited. The estimated habitual (3.2 mg/day) and recent (1.4 mg/day) intakes of isoflavones in women of our study were higher than those reported in previous studies. In postmenopausal White women who participated in the Framingham study, with less than 1 mg/d of isoflavone intakes [31]. Women who participated as controls in a study of ovarian cancer, consumed in average 1.8 mg/day of isoflavones [32], similar to our participants. 

Compared to Asian women [33], mean consumption of isoflavones in our study was approximately 8 times smaller. In terms of total lignans and coumestrol, our results from the FFQ were similar to the 1 mg/day and 1.4 mg/day, found in the Bandera et al. study [32]. Naringenin and quercetin were the most consumed flavonoids in our study and their main dietary sources were citrus fruits and onion, respectively.

Overall, our results indicate that the intake of dietary isoflavones exceeded consumption of lignans, which is contrary to the results reported by previous studies in Western diets [34,35]. This discrepancy could be due to the use of more individual phytoestrogens that were added for the assessment by group. Another reason could be the continuous use of soy protein, soy isolated, and soy flour as food additives in the manufacture of soy-based cereal, frozen desserts, energy bars, and particularly meat substitutes [36]. The FFQ included foods such as “Maizoro” cereal, commercial bread, breading, instant soups, and soy beverages, among others, that contributed to the estimates of isoflavone intakes. 

Some studies have evaluated the relationship between estimates of dietary intake and urinary excretion of lignans. As in our study, in Australian women, no association between habitual lignan excretion and dietary intake was found for the FFQ [37]. This is likely due to the wide variety of foods containing lignans, making estimations of lignan intake a challenge. The lack of analysis of the different types of lignans in the Western diet contributed to the underestimation of dietary lignans intake in our study, using the FFQ. The lignan or isoflavone concentrations can vary in the same food according to location, variety, crop season, and processing methods [38,39]. Therefore, measuring dietary intakes of phytoestrogens using a food-intake instrument and food composition databases is complicated and may not fully capture the intake of these compounds. Establishing a biomarker of phytoestrogen intake through the present study means that dietary intake can be estimated reasonably accurately from analysis of a 12 h urine collection.

This is the first study on the daily intake of phytoestrogens and their correlation with urinary excretion of these compounds in Mexican women. In a recent study from Northwest Mexico, the authors evaluated the use of serum phytoestrogens as a biomarker of phytoestrogen intake, and found correlations for some individual compounds, such as naringenin, luteolin, genistein, enterolactone, coumestrol, and resveratrol [19]. However, no correlations were found for groups or for total phytoestrogens. We found correlations between recent intakes and urinary levels for total phytoestrogens, and for groups of isoflavones, lignans, and flavonoids, as well as for genistein and naringenin as individual compounds. 

In a study that evaluated serum and urine as biomarkers of intake of phytoestrogens in the general US population, the authors discussed that the concentrations of phytoestrogens were lower in serum than in urine, which points to the fast clearance of these compounds from the body [40]. The time of the collection of the blood sample is an important variable for the determination of phytoestrogens because of their short half-lives. Isoflavones, in the form of glucuronic, predominate in urine, and half-life is approximately 7 to 10 h [41]. Enterolignans appear in circulation approximately 8 to 10 h after the ingestion of lignans derived from plants. In contrast, lignans derived from plants are in blood circulation after 2 h of consumption but their concentrations are lower than those of enterolignans [42]. Thus, urine collections may be more useful for the complete estimation of lignan and isoflavone levels than blood samples. The authors from the study previously mentioned mentioned that the high correlation observed for urinary and serum levels of phytoestrogens validates that noninvasive collection techniques, such as those used for urine, can be used to assess phytoestrogen exposure [40].

Another study in México estimated the dietary intake of phytoestrogens [18]. The authors applied an adapted FFQ to measure the consumption of flavonoids (flavonols, flavones, and flavanols), lignans (secoisolariciresinol, matairesinol, lariciresinol, and pinoresinol), and coumestrol. Only the intake of coumestrol was similar to that obtained in our study. The same estimations were made in a study of Torres-Sánchez et al. [43]. The differences in the intake of phytoestrogens between these two studies and our research could be due to the fact that they used different food composition databases. 

In addition to dietary intake, metabolism by intestinal bacteria can also influence an individual’s urinary levels of phytoestrogens, especially equol, which is transformed from daidzein by gut microflora [44]. The concentrations of equol that we found in the urine samples from the participants in our study (median, 23.1 ng/mL) were higher than those found in women from Hanoi, Vietnam (19 ng/mL), and exceeded the values of women in Japan (1.4 ng/mL) and USA (2.5 ng/mL) [15]. However, daidzein concentrations were lower in our sample, although we found a significant correlation coefficient between urinary daidzein concentration and its metabolite equol. Diet may contribute to the ability to harbor equol-producing bacteria [45,46]; therefore, not all humans (30–60%) possess the gut flora that produces equol [47].

When we estimated concentration ratios of equol to daidzein in urine samples, as an indicator of conversion efficiency, significantly higher ratios were found for our samples (ratio equol/daidzein = 0.61), compared to samples from women in Hanoi, Vietnam (ratio = 0.53), and Japan (ratio = 0.002) [15]. These results indicate a more efficient biotransformation of daidzein into its metabolite equol by the Mexican women in our study. Equol has a higher estrogenic activity when compared to daidzein, and has been proposed as an important component of isoflavones for disease prevention [48]. Biotransformation of daidzein to equol has been proposed as a key factor in the protective effects of phytoestrogens against breast cancer [49,50]. Thus, our results indicate that the population in Mexico comprised “good” equol producers, and this phenotype could have epidemiological implications for the reduction of some chronic diseases, such as breast cancer risk. 

We also observed that the ratio of enterodiol to enterolactone, which can interconvert, was 0.25 (less than 1), indicating that enterolactone was excreted in higher amounts than enterodiol. This finding is in agreement with other human studies [40,51]. The bacterial synthesis of enterolactone occurs via dehydroxylation and demethylation of matairesinol. Enterolactone is also produced by oxidation of enterodiol, which is a product of secoisolariciresinol metabolism [52]. The biological activity of enterolactone and enterodiol is different; enterolactone is a more potent aromatase inhibitor and has approximately 10 times the estrogenic activity than enterodiol [53]. According to Liu et al. [54], enterodiol and enterolactone both have potent inhibitory effects on ovarian cancer, but enterolactone possesses a more effective anti-cancer capability and fewer side effects than enterodiol. Enterolactone also has inhibitory effects on growth and metastasis in human breast cancer [55]. Thus, differences in metabolism and exposure of these lignans may be of physiological importance in cancer prevention. 

On the other side, variations between the estimated intake and the urinary levels of individual or group phytoestrogens, especially when we used the FFQ, could be due to the difficulty in estimating phytoestrogen intakes. The FFQ, by definition, is a semiquantitative questionnaire with a trend toward the overestimation of intake. Methods of dietary assessment are estimations of intake and rely on the veracity or reported intakes. Therefore, the accuracy of dietary data is influenced by memory recall, body size, sex, age, and ethnicity, as well as psychosocial and behavioral factors [56,57,58,59].

Studies in developing countries have shown that energy intake is underreported [60,61]. In fact, underreporting intake of some foods is apparent in our study, since energy intake from the 24 h recall was low, 1473 kcal/day. According to Scagliusi et al. [62], individuals with a lower income might have greater difficulties in the reporting tasks. Body mass index is also an important variable. As obesity increases, especially in the female population, underreporting energy intake is more common. Irregular meal habits and low education have been also associated with the under-report of energy intake [63]. In a study in New Zealand, the authors detected 265 unreported foods (often snacks) as revealed by the use of wearable cameras [64].

According to the above, we might think that the under-report of energy intake may be due to the frequency of overweight and obese persons in our study population (37.3% and 38.8% respectively, according to body mass index). Low income and education are factors that could contribute to the underreporting of energy intakes, since 64% of women in our study were from a lower-income level and 58.6% had less than 9 years of schooling. Considering the possibility of the underestimation of intake in our study population, reported consumption of unhealthy foods that are rich in fat, high in sugar, or highly processed may be lower than habitual intake; thus, fruits and vegetables, which represents a source of phytoestrogens, may be overestimated. 

A limitation of our study is the use of an FFQ validated for women in the region and modified for the study, although not validated specifically for phytoestrogens. The food list increased in the questionnaire, which could lead to an overestimation of phytoestrogen intake. The collection of 12 h instead of 24 h urine may be another limitation of our study. We used 12 h urine since it is a less burdensome method, and the 24 h urine collection was not well accepted by potential participants. Therefore, women collected their urine samples during the night, and we picked them up in the early morning. As an argument, we may say that, in the study of Grace et al. [65], a spot urine was sufficient for obtaining good results. Because of unpredictable changes in urine flow during the day and since the synthesis and total elimination of creatinine is constant [66], we adjusted the amount of phytoestrogens in urine to the concentration of creatinine. It is desirable that we could have collected urine samples for one or more days in each season of the year to consider seasonal variations in intakes. This could have allowed us to obtain better correlations between phytoestrogens from urine and dietary phytoestrogens estimated from the FFQ, because they represent the usual diet.

An advantage of using urine analysis could mean that we can avoid cases of omission or the overestimation of intake among participants, as well as the problem of not finding a food in the food database. Our quantification method is sensitive and reflects a change due to exposure, since the technique has high specificity and sensitivity. Therefore, the determination of urine levels provides a more accurate and objective measure of intake. The evaluation of 16 individual phytoestrogens, as well as the fact that there is no other study on this subject in Mexican women, is another advantage of our study. The results of our study could motivate other researchers to validate intakes of specific phytoestrogens. 

## 5. Conclusions 

In conclusion, this study has shown that the urinary excretion of total phytoestrogens is significantly correlated with habitual dietary intake. In addition, recent intakes of individual, group, and total phytoestrogens were related to their urinary levels. Therefore, phytoestrogens in urine may be a useful biomarker for the intake of these compounds. The study has furthermore shown the importance of the inclusion of several different phytoestrogens when using total phytoestrogens as a biomarker for intake. The validity of this approach should be extensively investigated. The production of equol by Mexican women is a new avenue of research. This study provides the basis for future studies that look forward to identifying the role of phytoestrogens in reducing the risk of breast cancer and other chronic diseases in the Mexican population.

## Figures and Tables

**Table 1:** Isoflavone, coumestrol, and resveratrol intakes estimated by the FFQ in Mexican women (*n* = 100).

Phytoestrogens	Mean ± SD	Geometric Mean *	Value	Percentiles		
(µg/Day)		(95% CI)	Min–Max	25	50	75
Daidzein	1151 ± 3211	388.5 (283–533)	30.7–26,249	145.8	232.5	867.9
Genistein	1642 ± 5251	642 (452–912)	38.0–46,147	156.0	296.0	1023.7
Glicitein	14.8 ± 16.9	11.3 (9.5–13.4)	2.6–107.3	7.3	10.4	15.3
Biochanin A	310 ± 279	270.5 (236–310)	23.7–1420.6	105.9	234.8	471.5
Formononetin	53.0 ± 110.2	28.3 (22.6–35.5)	1.6–775.7	14.2	23.9	42.1
Equol	2.4 ± 1.9	1.9 (1.6–2.2)	0.2–13.6	1.1	1.9	3.16
Total isoflavones	3173 ± 8456	1168 (894–1526)	289–73,037	625.8	1025.4	2297.8
Coumestrol	1850 ± 1730	1569 (1333–1847)	96.6–8633.7	541.9	1361.3	2901
Resveratrol	17.4 ± 43.7	5.2 (3.6–7.4)	0.006–290.3	0.9	4.4	14.8

FFQ: Food Frequency Questionnaire; SD: Standard Deviation; 95% CI: 95% Confidence Interval; * Adjusted for energy intake by the residual method [25]. Total isoflavones consist of the sum of daidzein, genistein, glicitein, biochanin A, formononetin, and equol.

**Table 2:** Lignan, flavonoid, and total phytoestrogen intakes estimated by the FFQ in Mexican women (*n* = 100).

Phytoestrogens	Mean ± SD	Geometric Mean *	Value	Percentiles		
(µg/Day)		(95% CI)	Min–Max	25	50	75
Secoisolariciresinol	712.8 ± 1448	490.5 (386–623)	69.6–11,771	145.6	222.5	586.9
Matairesinol	20.1 ± 14.0	17.1(15.3–19.1)	3.9–97.3	11.4	17.2	25.1
Enterodiol	67.4 ± 37.6	62.5 (57.2–68.3)	16.4–222.3	42.3	61.8	80.9
Enterolactone	183.2 ± 115.2	156.7 (138–178)	49.9–662.7	102.2	158.9	227.6
Total lignans	1171 ± 1586	872.7 (742–1027)	240.9–13,335	439.9	754.9	1182.3
Naringenin	15,940 ± 16,582	12,429 (10,243–15,082)	737.2–111,747	4887.4	10,997	20,759
Luteolin	1555 ± 1355	1239 (1065–1441)	143.9–8772.0	670.2	1221.0	2019.4
Kaempferol	826.1 ± 685.4	625.5 (538–728)	120.0–4519.6	351.1	649.6	1018.3
Quercetin	13,023 ± 6912	11,353 (10,129–12,724)	1719.3–31,993	7873.9	11,472	17,865
Total flavonoids	31,344 ± 20,862	26,773 (23,600–30,373)	6527–139,335	15,617	28,101	39,288
Total phytoestrogens	37,557 ± 23,909	31,957 (28,322–36,059)	9016–159,239	20,716	32,716	48,554

FFQ: Food Frequency Questionnaire; SD: Standard Deviation; 95% CI: 95% Confidence Interval; * Adjusted for energy intake by the residual method [25]. Total lignans consist of the sum of secosiolariciresinol, matairesinol, enterodiol, and enterolactone. Total flavonoids consist of the sum of naringenin, luteolin, kaempferol, and quercetin. Total phytoestrogens consist of the sum of isoflavones, lignans, coumestrol, flavonoids, and resveratrol.

**Table 3:** Isoflavone, coumestrol, and resveratrol intakes estimated by the 24 h recall in Mexican women (*n* = 100).

Phytoestrogens	Mean ± SD	Geometric Mean *	Value	Percentiles		
(µg/Day)		(95% CI)	Min–Max	25	50	75
Daidzein	517 ± 2234	214 (157–289)	0.67–16,220	22.5	86.4	135.7
Genistein	775 ± 3508	262 (184–375)	0.58–26,450	24.17	70.1	173.8
Glicitein	9.46 ± 9.81	6.14 (5.06–7.45)	0.485–57.9	3.01	5.24	14.7
Biochanin A	57.9 ± 81.1	37.8 (29.0–49.2)	0.02–520	4.62	29.9	76.4
Formononetin	19.8 ± 25.6	14.2 (10.8–18.6)	0–159.4	1.38	4.28	31.3
Equol	1.13 ± 1.52	0.67 (0.49–0.92)	0–6.55	0	0.44	1.66
Total isoflavones	1380 ± 5710	567 (423–761)	3.3–40,789	116.5	271.6	441.5
Coumestrol	403 ± 1255	186 (132–264)	0.09–11,643	1.41	3.65	433.4
Resveratrol	2.13 ± 21.1	2.91 (2.15–3.94)	0–211.2	0	0	0

SD: Standard Deviation; 95% CI: 95% Confidence Interval; * Adjusted for energy intake by the residual method [25]. Total isoflavones consist of the sum of daidzein, genistein, glicitein, biochanin A, formononetin, and equol.

**Table 4:** Lignan, flavonoid, and total phytoestrogen intakes estimated by the 24 h recall in Mexican women (*n* = 100).

Phytoestrogens	Mean ± SD	Geometric Mean *	Value	Percentiles		
(µg/Day)		(95% CI)	Min–Max	25	50	75
Secoisolariciresinol	307 ± 1767	65.7 (50.0–86.4)	9.12–16,938	31.83	52.2	81.3
Matairesinol	16.0 ± 45.2	9.61 (7.83–11.8)	0.58–438	4.14	7.24	13.9
Enterodiol	18.7 ± 23.0	15.2 (12.5–18.5)	0–133.6	1.99	11.6	26.3
Enterolactone	67.6 ± 83.4	53.4 (42.9–66.4)	0–435.5	8.59	41.7	88.7
Total lignans	409.1 ± 1781	162.4 (130–204)	15.6–17,001	75.0	130	212
Naringenin	5352 ± 18,834	1356 (901–2040)	0–151,179	0	196	1191
Luteolin	471 ± 1081	157 (108–228)	0–6379.3	5.77	46.5	330.8
Kaempferol	476 ± 1274	327 (247–434)	0–8224.2	29.94	116.9	255.9
Quercetin	8170 ± 8954	5202 (4090–6616)	0–56,088	1514	5851	11,277
Total flavonoids	14,471 ± 21,693	8432 (6578–10,807)	0–160,534	2775	8467	18,208
Total Phytoestrogens	16,663 ± 22,858	10,838 (8757–13,414)	54–161,360	3591	9842	20,258

SD: Standard Deviation; 95% CI: 95% Confidence Interval; * Adjusted for energy intake by the residual method [25]. Total lignans consist of the sum of secosiolariciresinol, matairesinol, enterodiol, and enterolactone. Total flavonoids consist of the sum of naringenin, luteolin, kaempferol, and quercetin. Total phytoestrogens consist of the sum of isoflavones, lignans, coumestrol, flavonoids, and resveratrol.

**Table 5:** Urinary concentrations of phytoestrogens during 12-h urine collection period (*n* = 100).

Phytoestrogens	Mean ± SD	Geometric Mean *	Percentiles			Geometric Mean *
(µg/L)		(95% CI)	25	50	75	µg/g Creatinin (95% CI)
Daidzein	117 ± 236	31.2 (21.9–44.4)	10.9	38.1	103	56.0 (39.7–79.1)
Genistein	49.2 ± 101	17.4 (12.5–24.1)	4.62	18.4	48.8	30.9 (22.6–42.5)
Glicitein	34.5 ± 88.3	25.5 (16.6–39.4)	0	0	25.8	43.5 (28.7–66.1)
Biochanin A	1.25 ± 1.04	1.02 (0.89–1.17)	0.68	1.08	1.50	1.86 (1.59–2.18)
Formononetin	2.42 ± 1.71	2.31 (2.05–2.61)	1.49	2.16	3.28	4.19 (3.72–4.72)
Equol	42.4 ± 120	29.9 (25.3–35.5)	14.2	23.1	40.3	51.2 (44.0–59.4)
Secoisolariciresinol	3.45 ± 5.73	2.89 (2.36–3.55)	0.69	1.91	5.13	5.49 (4.39–6.86)
Matairesinol	0.64 ± 2.07	1.10 (0.63–1.90)	0	0	0.11	1.82 (1.01–3.26)
Enterodiol	2.86 ± 12.9	10.5 (5.93–18.7)	0	0	0	19.7 (11.9–32.6)
Enterolactone	64.9 ± 66.8	41.7 (33.5–51.9)	24.8	46.7	82.3	75.9 (59.8–96.4)
Coumestrol	1.24 ± 1.09	1.10 (0.91–1.33)	0.55	1.01	1.72	2.07 (1.71–2.50)
Resveratrol	11.9 ± 108	1.32 (0.89–1.96)	0	0.23	1.72	2.51 (1.69–3.73)
Luteolin	6.72 ± 32.5	2.57 (2.12–3.10)	1.40	2.38	4.31	4.61 (3.85–5.53)
Kaempferol	25.8 ± 54.9	12.9 (9.67–17.4)	2.13	10.4	26.1	22.9 (17.2–30.8)
Naringenin	135 ± 213	60.1 (46.3–78.1)	26.2	64.8	127	108 (83.2–140)
Quercetin	2.96 ± 7.14	2.15 (1.77–2.60)	0.71	1.83	3.34	3.92 (3.25–4.73)
Total Phytoestrogens	502 ± 580	336 (284–398)	213	306	5175	603 (511–713)

SD: Standard Deviation; 95% CI: 95% Confidence Interval; * Adjusted for energy intake by the residual method [25]. Total phytoestrogens consist of the sum of total isoflavones, total lignans, coumestrol, total flavonoids, and resveratrol.

**Table 6:** Corrected correlation coefficients between urinary metabolites and dietary estimates of phytoestrogen intake (Habitual-FFQ and Recent-24 h recall).

Phytoestrogen	Corr CC	*p*-Value	Corr CC	*p*-Value
	Habitual (µg/Day) vs. Urine (µg/g Creatinine)		Recent (µg/Day) vs. Urine (µg/g Creatinine)	
Daidzein	−0.024	0.840	0.099	0.420
Genistein	−0.034	0.784	0.374	0.002
Equol	0.126	0.283	0.079	0.500
Glicitein	0.055	0.657	0.120	0.330
Biochanin A	0.012	0.923	0.160	0.190
Formononetin	−0.024	0.841	0.170	0.160
Total Isoflavones	0.002	0.983	0.460	0.0001
Secoisolariciresinol	0.009	0.934	0.230	0.060
Matairesinol	0.079	0.524	0.028	0.810
Enterolactone	−0.035	0.775	0.067	0.580
Enterodiol	−0.168	0.171	0.130	0.270
Total lignans	−0.111	0.359	0.550	0.000
Naringenin	0.069	0.571	0.620	0.000
Luteolin	−0.104	0.398	−0.200	0.090
Kaempferol	−0.084	0.496	0.040	0.690
Quercetin	0.154	0.212	0.027	0.820
Total Flavonoids	0.055	0.653	0.240	0.043
Coumestrol	−0.035	0.800	0.120	0.320
Resveratrol	0.337	0.005	0.005	0.960
Total Phytoestrogens	0.730	0.000	0.410	0.0007

FFQ: Food Frequency Questionnaire; Corr CC: Corrected correlation coefficient for intra-interindividual variation. Total isoflavones consist of the sum of daidzein, genistein, glicitein, biochanin A, formononetin, and equol. Total lignans consist of the sum of secosiolariciresinol, matairesinol, enterodiol, and enterolactone. Total flavonoids consist of the sum of naringenin, luteolin, kaempferol, and quercetin. Total phytoestrogens consist of the sum of isoflavones, lignans, coumestrol, flavonoids, and resveratrol.
